# Genomic mosaicism in the pathogenesis and inheritance of a Rett syndrome cohort

**DOI:** 10.1038/s41436-018-0348-2

**Published:** 2018-11-08

**Authors:** Qingping Zhang, Xiaoxu Yang, Jiaping Wang, Jiarui Li, Qixi Wu, Yongxin Wen, Ying Zhao, Xiaoying Zhang, He Yao, Xiru Wu, Shujie Yu, Liping Wei, Xinhua Bao

**Affiliations:** 1 Department of Pediatrics, Peking University First Hospital, Beijing, China; 2 Center for Bioinformatics, State Key Laboratory of Protein and Plant Gene Research, School of Life Sciences, Peking University, Beijing, China; 3 Peking-Tsinghua Center for Life Sciences, Academy for Advanced Interdisciplinary Studies, Peking University, Beijing, China; 4 Department of Neurology, Harbin Children’s Hospital, Harbin, China

**Keywords:** Rett syndrome, *MECP2*, somatic mosaicism, paternal germline mosaicism

## Abstract

**Purpose:**

To determine the role of mosaicism in the pathogenesis and inheritance of Rett and Rett-like disorders.

**Methods:**

We recruited 471 Rett and Rett-like patients. Panel-sequencing targeting *MECP2*, *CDKL5*, and *FOXG1* was performed. Mosaicism was quantified in 147 patients by a Bayesian genotyper. Candidates were validated by amplicon sequencing and digital PCR. Germline mosaicism of 21 fathers with daughters carrying pathogenic *MECP2* variants was further quantified.

**Results:**

Pathogenic variants of *MECP2/CDKL5*/*FOXG1* were found in 324/471 (68.7%) patients. Somatic *MECP2* mosaicism was confirmed in 5/471 (1.1%) patients, including 3/18 males (16.7%) and 2/453 females (0.4%). Three of the five patients with somatic *MECP2* mosaicism had mosaicism at MECP2-Arg106. Germline *MECP2* mosaicism was detected in 5/21 (23.8%) fathers.

**Conclusion:**

This is the first systematic screening of somatic and paternal germline *MECP2* mosaicism at a cohort level. Our findings indicate that somatic *MECP2* mosaicism contributes directly to the pathogenicity of Rett syndrome, especially in male patients. MECP2-Arg106 might be a mosaic hotspot. The high proportion of paternal germline *MECP2* mosaicism indicates an underestimated mechanism underlying the paternal origin bias of *MECP2* variants. Finally, this study provides an empirical foundation for future studies of genetic disorders caused by de novo variations of strong paternal origin.

## INTRODUCTION

Rett syndrome (RTT) is a neurodevelopmental disorder affecting females almost exclusively, and the majority of patients are sporadic. Methyl-CpG-binding protein 2 (*MECP2*) is the main causative gene of RTT; 95% of classical RTT cases were found to be caused by *MECP2* pathogenic variants.^1^ Cyclin-dependent kinase-like 5 (*CDKL5*) and Forkhead box protein G1 (*FOXG1*) are responsible for the early seizure variant and congenital variant of atypical RTT, respectively.^2,3^ However, for a subset of patients with RTT and RTT-like phenotypes, no pathogenic variants have been identified in *MECP2*, *CDKL5*, or *FOXG1*. Recently, postzygotic variants have drawn increasing attention in the field of disease genetics.^4^ More than 40 nontumorous monogenetic diseases have been discovered to arise from mosaic variants of relevant genes.^5,6^ However, there have been only a few studies of mosaicism in RTT.^7,8^ Somatic *MECP2* mosaicism has been described in sporadic RTT cases, for which variants have been identified and estimated by Sanger sequencing.^8–15^ However, the epidemiology of *MECP2* mosaicism in RTT cohorts, mutant allelic fractions (MAFs), and the relationship between these factors and the severity of RTT are poorly understood.

Several studies have reported that 94–96% of *MECP2* variants in sporadic cases of RTT were of paternal origin.^16,17^ However, the mechanisms underlying RTT in these cases remain unclear. Recent studies of Apert syndrome, Crouzon syndrome, and Pfeiffer syndrome found that genomic mosaicism in paternal gametes is responsible for the disease in their offspring. These results might also explain the significant paternal bias in the origin of “spontaneous mutations”^18^ for diseases such as Apert syndrome. For some diseases, a selection advantage for the mutant cells of fathers has been identified, and this advantage contributes to the accumulation of pathogenic variants in their offspring.^18,19^ However, with regard to RTT, there is limited knowledge about germline mosaicism in the fathers of disease-affected individuals. Germline *MECP2* mosaicism has been identified in only one man with two RTT daughters, who were half-sisters.^20^ The occurrence rate of *MECP2* mosaicism in the parents of RTT patients, especially in their germline cells, and the variance of MAFs between different tissues from the same individual remains unknown. Therefore, this study was conducted to investigate the role of somatic and paternal germline mosaicism in the pathogenicity and inheritance of RTT spectrum disorders.

## MATERIALS AND METHODS

**Fig. 1:**
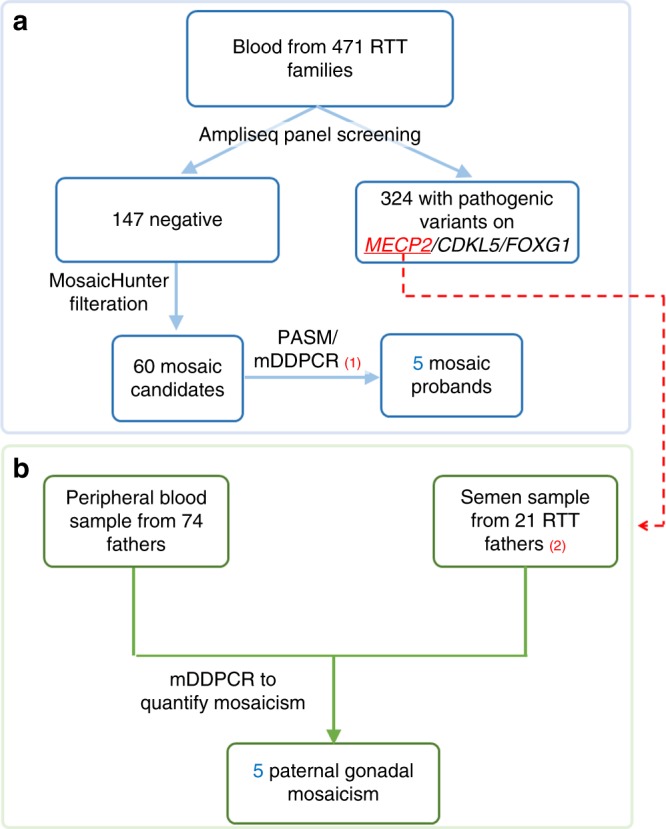
**Overview of the subjects involved in this study.** (1) Of the 60 probands with candidate mosaic variants, 13 were validated by PASM, 31 were validated by mDDPCR, and 16 were validated by PASM and mDDPCR. (2) Among the 21 fathers, 10 subjects also contributed urine, saliva, hair, and buccal samples. *mDDPCR* microdroplet digital polymerase chain reaction, *PASM* Personal Genome Machine amplicon sequencing of mosaicism, *RTT* Rett syndrome.

An overview of the workflow is shown in Fig. 1. Details are described below.

### Capture panel sequencing and multiplex ligation-dependent probe amplification in the RTT and RTT-like cohorts

#### Subjects

Patients with typical/atypical RTT and RTT-like syndrome were enrolled. The diagnostic criteria for typical and atypical RTT were adopted from Neul.^21^ RTT-like syndrome refers to a clinical manifestation that does not completely meet the criteria for RTT, but which shares the main features of RTT, including psychomotor retardation with or without regression, stereotypic hand movements, and other autism-like behaviors. In total, 471 patients (453 females and 18 males; Supplemental material, Table S1) were recruited from the child neurology units of Peking University First Hospital from January 2000 to January 2018.

This study was approved by the Institutional Review Board at Peking University (IRBPU) and the Ethics Committee of Peking University First Hospital under approval code IRB00001052-11087. Written informed consent forms were obtained from the parents of each subject. All methods used in this study were performed in accordance with the relevant guidelines and regulations of the IRBPU.

Genomic DNA from peripheral blood was extracted using a salting-out procedure. Genomic DNA from hair follicles, buccal swabs, saliva, and urine was extracted using a QIAamp® DNA Micro Kit (#56304, Qiagen, Germany).

#### Targeted sequencing panels

An AmpliSeq capture panel (Ion Torrent) targeting the whole genomic region and regulatory sequences of *MECP2*, *CDKL5*, and *FOXG1* was designed. A sample of genomic DNA (10 ng, approximately 3000 genome copies) was used to prepare the library using a standard Ion AmpliSeq library preparation protocol (pub. no. MAN0006735) with some modifications. Illumina adapters were used to maximize throughput and minimize sequencing cost. Two rounds of polymerase chain reaction (PCR)-based enrichment were performed to add indexes to the libraries. The pooled library was sequenced on an Illumina HiSeq 2500 platform (CA, USA) to generate 100 bp paired-end reads. The average read depth was approximately 1000×. Reads were aligned to a human reference genome (GRCh37/hg19) with the Burrows–Wheeler Aligner (BWA) mem algorithm in the Burrows–Wheeler Aligner software.^22^ The workflow for data analysis followed the best practice workflows of the Genome Analysis Toolkit (GATK) 3.2–2. The methods used for variant annotation were described in a previous publication.^23^

#### MLPA

Multiplex ligation-dependent probe amplification (MLPA) (SALSA MLPA kit P015 *MECP2*, MRC-Holland, Amsterdam, Holland) was performed to detect large deletions or duplications of the *MECP2* gene as previously described.^24^ MLPA products were separated and analyzed using an ABI Prism 3100 Genetic Analyzer and Gene Scan software according to the manufacturer’s recommendations.

### *MECP2* mosaicism screening

#### Subjects

One hundred forty-seven RTT and RTT-like patients (Supplemental material, Table S2) without detectable *MECP2/CDKL5/FOXG1* variants were subjected to somatic mosaic screening. The next-generation sequencing (NGS) data of the subjects were analyzed using MosaicHunter. Candidate mosaic variants were selected and validated by microdroplet digital PCR (mDDPCR).

Seventy-four fathers were subjected to somatic mosaic detection by mDDPCR. DNA from peripheral blood was measured (Supplemental table S6). Each of the tested fathers had at least one daughter with RTT who was confirmed to possess a de novo pathogenic *MECP2* variant.

Next, germline *MECP2* mosaic variants were tested in 21 available fathers (in addition to the 74 fathers mentioned above) who volunteered to donate a semen sample. Ten of the 21 fathers also donated additional sample material, including hair follicles, buccal swabs, saliva, and urine (Supplemental material, Table S3). mDDPCR was applied to test for mosaicism. Sperm from two males (ACC1-sp and ACC4-sp) without a family history of RTT was used as negative control material. mDDPCR was used to quantify *MECP2* mosaic variants.

#### DNA isolation

Sperm samples were purified with a PureSperm 40/80 assay (Nidacon, Sweden), and four different semen components were collected separately, including Sperm, Layer 2, Layer 1, and Others (Fig. 4a). Genomic DNA from the four semen components was extracted using a phenol–chloroform extraction method (ref. ^25^).

#### Filtration for mosaic candidates by MosaicHunter

Reads generated from panel sequencing (see section on MLPA) were realigned to human reference genome GRCh37/hg19 by BWA mem. Postalignment processing, indel realignment, and base quality recalibration were carried out following the GATK 3.2–2 best practice workflows. As described previously, the processed reads were filtered by a simplified Bayesian model (https://github.com/Yyx2626/yyxMosaicHunter) to quantify the candidate mosaic variants. The MAF threshold for the lower bound of the 95% Bayesian confidence interval (CI) was 0.5% for the reference homozygous genotype. The MAF threshold for the heterozygous genotype was 45.0–55.0% (ref. ^26^).

#### PGM amplicon sequencing of mosaicism

Personal Genome Machine amplicon seuencing of mosaicism (PASM) was used to validate candidate mosaic variants that were not located on mutant hotspots. Targeted PCR amplification was used to capture a region of 400 base pairs around the candidate genome position. The amount of DNA used for amplification was 20 ng (approximately 6000 genome copies). The primers are listed in Table S4. An amplicon library was prepared using an Ion Xpress^TM^ Plus Fragment Library Kit and sequenced on an Ion Torrent Personal Genome Machine (PGM) using Ion 318 V2 chips (ThermoFisher). The average read depth for PASM was approximately 12,000×. A hierarchical Bayesian model was used to calculate MAFs with maximum a posteriori estimation and 95% Bayesian confidence intervals (CIs) (https://github.com/Yyx2626/yyxMosaicHunter). The MAF threshold for the lower bound of the reference homozygous variants was 0.5%. The MAF threshold for the heterozygous variants was 40.0–60.0%. According to our previous benchmark tests, the validation sensitivity is 0.85, while the specificity is 0.92 (ref. ^25^).

#### Measurement of allelic fractions by mDDPCR

mDDPCR with single-molecule resolution was used to accurately measure MAFs. To avoid potential contamination of low-fraction mutant alleles, DNA from multiple tissue types was sheared separately. Ultraviolet treatment was carried out after shearing DNA from each proband. Next, mDDPCR analysis was carried out for the absolute quantification of MAFs.^25^ TaqMan genotyping assays targeting ten mutational hotspots and two nonhotspot sites (Supplemental material, Table S5) in *MECP2* were designed. The mutant allele was labeled with FAM fluorophore, whereas the wild-type allele was labeled with VIC fluorophore (P/N:4331349, Applied Biosystems, IDs provided in Table S5). Genotyping quantitative PCR (qPCR) experiments were first performed on a StepOne Plus real-time system (Applied Biosystems by ThermoFisher) to test the performance of the assays. The validated genotyping system was subjected to the downstream digital PCR reactions. Droplet emulsions were generated from a Raindrop Source droplet generator. PCR amplification was carried out with a controlled temperature ramp of 0.5 °C/s. Fluorescent droplets were detected on a RainDrop^TM^ Sense droplet detector. RainDrop Analyst V3 software was used for data analysis. Ninety-five percent confidence intervals of MAFs were calculated with a binomial distribution. The detection limit of mDDPCR was 10^−4^ alternative allele/total genomic copies.

## RESULTS

### Variant spectrum of *MECP2*, *CDKL5*, and *FOXG1* in a Chinese RTT cohort

Variants in *MECP2*, *CDKL5*, or *FOXG1* were detected in 68.7% (324/471) of the patients. Among the patients, 315 had *MECP2* pathogenic variants, 5 had *CDKL5* pathogenic variants, and 4 had *FOXG1* pathogenic variants (Supplemental table S7).

### Somatic *MECP2* mosaicism in RTT probands

**Fig. 2:**
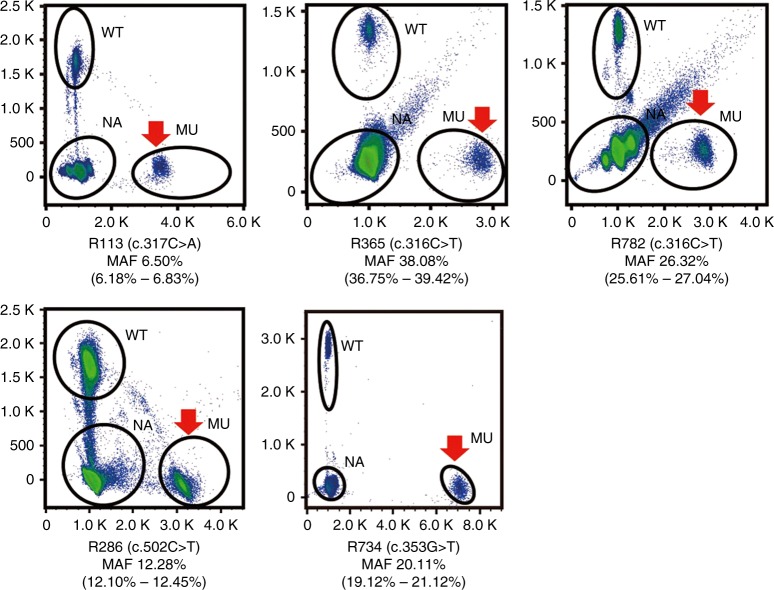
***MECP2* mosaicisms were identified from the blood samples of five patients by microdroplet digital polymerase chain reaction (mDDPCR).** Mosaic variants are clearly demonstrated on the flow cytometry scatter plots of the mDDPCR results under the red arrow near “MU” at the bottom right corner. The 95% confidence intervals (CIs) for the mutant allele fraction (MAF = MU/[MU + WT]) were calculated under a binomial distribution. *MU* signals from mutant alleles, *NA* signals from droplets that did not contain target sequences and thus could not be amplified, *WT* signals from wild-type alleles.

A mosaic variant of *MECP2* was detected in 5 patients (1.1%, 5/471), including 3 males (16.7%, 3/18) and 2 females (0.4%, 2/453). The somatic mosaicism rate observed in male patients was significantly higher than that in females (odds ratio = 43.47, *p* = 0.0004492 by a two-tailed Fisher’s exact test). The MAFs ranged from 6.50% to 38.08% (Fig. 2). Among the patients with *MECP2* mosaicism, 4 patients (2 males and 2 females) were diagnosed with typical RTT, whereas 1 (male) patient was diagnosed with RTT-like syndrome (Supplemental material, Table S8).

**R113.** Patient R113 is a boy who was 4 years and 4 months of age at the time of the study and had developmental delay. The patient could raise his head at the age of 5 months, sit unsupported at 7 to 8 months, and walk with aid at 1 year. He was capable of walking unstably and could speak 1 to 2 words at 4 years and 4 months of age. He had some hand skills, such as grabbing larger objects, but his fine motor skills were poor. Although delayed, there was no significant developmental regression. He was diagnosed with RTT-like syndrome. A mosaic variant of *MECP2*, c.317G>A, p.(Arg106Gln), was found, with a MAF of 6.50%.

There was a female with a heterozygous variant at the same genomic position as patient R113 in our cohort. At the time of the study, she was 3 years old with a typical RTT phenotype. She could raise her head at 4 months, sit alone at 9 months, and walk at 20 months. Her gait was unstable at 3 years of age. She could speak single words at 3 years old. Repetitive hand movements occurred at 20 months, and then hand skills were gradually lost. In comparison with this female patient, the symptoms of patient R113 were slightly milder in severity.

**R365.** Patient R365 is a girl with typical RTT who was 2 years and 4 months of age at the time of the study. She could raise her head at 2 months, sit alone at 8 months, and walk and speak at 16 months. Regression started at 17 months, at which point she gradually lost her acquired language skills. Repetitive hand movements were observed at 19 months of age, after which purposeful hand skills were lost. Seizures occurred at 19 months of age. She was found to possess *MECP2* mosaic variant c.316C>T, p.(Arg106Trp) with a MAF of 38.08%.

**Fig. 3:**
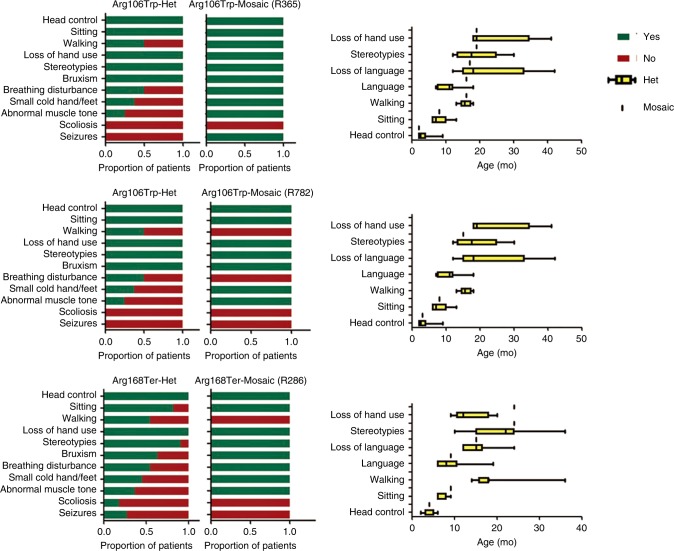
**Comparison of the phenotype descriptions of mosaic patients with those of heterozygous patients carrying variants at the same genomic position.**

Heterozygous pathogenic variants of *MECP2* at this genomic position were identified in 16 female patients in our cohort. With gross motor development, language learning, the onset age of stereotypies, and the age of regression taken into consideration, there was no significant difference between patient R365 and the heterozygous females in this study (Fig. 3).

**R782**. Patient R782 was a boy who was 2 years and 7 months of age at the time of the study. He was found to possess *MECP2* mosaicism c.316C>T, p.(Arg106Trp) with a MAF of 26.3%, and he presented with typical RTT. The patient achieved head control at 3 months, sat alone at 8 months, and was incapable of walking independently at the time of the study. Hand clapping and wringing occurred at 15 months of age. Psychomotor regression was noticed at 1.5 years of age. He had neither purposeful hand skills nor the capacity for meaningful language.

In comparison with 16 females carrying heterozygous variants at the same genomic position, stereotypic hand movement occurred earlier in the mosaic male patient (15 months vs. 20 months). In addition, the male mosaic patient never acquired hand skills or language skills (Fig. 3). Overall, the phenotype of the mosaic male patient was slightly more severe than those of heterozygous females with the same mosaic variant.

**R286**. Patient R286 was a girl who was 3 years and 1 month of age at the time of the study and was diagnosed with typical RTT. Her developmental milestones were nearly normal, with head control at 4 months and sitting at 9 months, but she was incapable of walking. Simple language started at 9 months with words such as *mama*. Psychomotor regression was noticed at 15 to 16 months old. Frequent hand stereotypies and loss of hand skills occurred at 19 months old. Bruxism was also noticed. *MECP2* mosaic variant c.502C>T, p.(Arg168Ter) was identified in this patient with a MAF of 12.28%.

A *MECP2* heterozygous variant at the same site was identified in 11 females in our cohort. The phenotype of patient R286 and those of the patients with the heterozygous *MECP2* variant were compared (Fig. 3). The onset age of hand stereotypies and loss of hand use were delayed in the mosaic patient in comparison with the heterozygous patients. However, with regard to developmental milestones, including raising her head, sitting, and walking, she lagged behind the heterozygous patients.

**R734.** Patient R734 was a 2.5-year-old boy at the time of the study. He could raise his head at 4 months of age, sit at 1 year, and walk with assistance at 2.5 years. Simple language started at 11 months and gradually disappeared after 13 to 14 months of age. Stereotypic hand movement was noticed at 1 year of age, after which hand skills regressed. Typical RTT was diagnosed. *MECP2* mosaicism c.353G>A, p.(Gly118Val) was identified with a MAF of 20.11%. This variant was novel, and it was predicted as damaging by MutationTaster, PolyPhen-2, and SIFT. The same variant was not identified in the parents of the patient.

### Somatic and germline *MECP2* mosaicism in fathers

A total of 74 paternal peripheral blood samples were subjected to PASM and/or mDDPCR. However, no somatic mosaic variant was identified (Supplemental Figure S1, and Table S6).

**Fig. 4:**
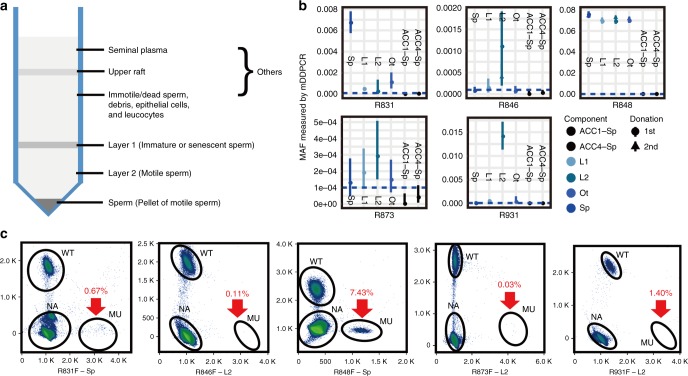
**Five fathers carrying germline mosaic variants of** ***MECP2***. (**a**) Schematic diagram of the semen components that were separated using the PureSperm 40/80 assay. (**b**) Distribution of mosaicism in each component of the semen samples. Sp vital sperm, L1 the layer containing immature or senescent sperm, L2 the layer containing motile sperm, Ot other components such as seminal plasma, immotile/dead sperm, epithelial cells, leukocytes, and semen that failed to liquefy. ACC1-sp and ACC4-sp were sperm donated by asymptomatic males, which served as control samples. *MAF* (multiple allele fraction), *mDDPCR* (microdroplet digital polymerase chain reaction), *MU* (mutant), which means signals from mutant alleles, *NA* (not avaliable), which means signals from droplets that did not contain target sequences and thus could not be amplified, *WT* (wild-type), which means signals from wild-type alleles. (**c**) Mosaic variants are clearly demonstrated on the flow cytometry scatter plots of the mDDPCR results under the red arrow near “MU” at the bottom right corner. The 95% confidence intervals (CIs) for the mutant allele fraction (MAF = MU/[MU + WT]) were calculated under a binomial distribution.

Germline mosaic variants were found in 5 fathers (5/21, 23.8%). Three fathers possessed the c.502C>T, p.(Arg168Ter) variant, one possessed the c.880C>T, p.(Arg294Ter) variant, and one possessed the c.806delG, p.(Gly269Alafs*20) variant. The MAFs ranged from 0.03% to 7.55% (Table 1). As mentioned above, each semen sample was separated into four different components (Fig. 4a). For three fathers (R846F, R873F, and R931F), mosaicism was only detectable in Layer 2. For subject R831F, mosaicism was detectable in Sperm and Others. For subject R848F, mosaicism was detectable in all four layers (Fig. 4b, c). The sperm viability of four fathers was tested. One father (R831F) was diagnosed with asthenospermia (25%, 3/4), while one father (R848F) nearly met the diagnostic criteria for asthenospermia (Table 1). Subject R831F has two children (a RTT daughter and a normal son), whereas R848F has one child. The children of subjects R831F and R848F were all born after natural conception.

**Table 1:** Sperm morphology and kinetics of fathers with *MECP2* mosaicism

		R831F	R846F	R848F	R873F	R931F
DNA Variation		c.502C>T	c.502C>T	c.806delG	c.880C>T	c.502C>T
AA change		p.Arg168Ter	p.Arg168Ter	p.Gly269fsTer	p.Arg294Ter	p.Arg168Ter
MAFs		0.67%	0.11%	7.55%	0.03%	1.40%
Other samples		Negative	Negative	0.43% in saliva, 0.28% in blood	Negative	Negative
Semen volume (mL) (ref: ≥2 mL)		3	-	3	3	1
Sperm density (million/mL) (ref: ≥ 20 million/mL)		3.29	-	27.89	82	115.55
Sperm motility (ref: a ≥ 25% or a + b ≥ 50%)	a	10.34%	-	0%	11%	52.19%
	b	3.45%	-	26.09%	31%	18.08%
	c	3.45%	-	30.44%	10%	12.83%
	d	82.76%	-	43.48%	48%	16.91%
Teratospermia (ref: normal sperm≥30%)	Normal	0%	-	13.04%	8.29%	Unknown
	Head deformity	100%	-	86.96%	91.71%	Unknown
	Neck deformity	0%	-	0%	14.63%	Unknown
	Tail deformity	0%	-	0%	1.95%	Unknown
	Other deformity	0%	-	0%	0%	Unknown
(a + b)*(Density)*(volume) (ref: ref: ≥ 20 million/mL)		1.36	-	21.83	103.32	81.20
Conclusion		Asthenospermia	NA	Close to asthenospermia	Normal	Normal
Number of offspring		2	1	1	1	1

The diagnostic criteria for asthenospermia were as follows: total number of sperm with forward motility less than 20 million/mL or total proportion of sperm with normal morphology lower than 4%. Abnormal indexes are marked in red.

*AA* amino acid, *MAF* multiple allele fraction, *NA* not available, *Ref* reference range.

mDDPCR was performed on blood, hair, buccal, saliva, and urine samples. However, with the exception of R848F, who possessed mosaicisms in blood and saliva with MAFs of 0.28% and 0.43%, respectively, these tests were negative in all subjects (Supplemental material, Figure S2).

## DISCUSSION

In this study, we identified *MECP2* somatic mosaic variants in 5 RTT patients (1.1%, 5/471). This study is the first in which mosaicism screening was carried out at a cohort level in RTT patients. Before this study, mosaicism was only described in 8 RTT patients (6 males and 2 females) with MAFs ranging from 10% to 37% (refs. ^10,15^). First, we estimated the contribution rate of *MECP2* mosaicism in the pathogenesis of RTT, especially in those without detectable *MECP2/CDKL5/FOXG1* pathogenic variants. We found that the pathogenic MAF could be as low as 6.50% in blood. Such low MAFs cannot be detected with conventional Sanger sequencing or low-coverage NGS methods, so a more precise and sensitive method of mosaicism detection is required. Although somatic mosaic variants detected in blood samples might not directly reflect the corresponding MAF in the brain, recent quantification by Huang et al. and mathematical modeling by Ye et al.^27,28^ suggested that mosaic variants with higher MAFs are more likely to be shared among different tissues. Thus, postzygotic mosaic variants might exist in the brains of patients with detectable mosaic variants in the blood. For the remaining patients without heterozygous or mosaic pathogenic variants, the etiology remains unclear. Therefore, new causative genes or somatic variants with lower MAFs may be the reason. We plan to perform genome sequencing on them, hoping to identify new causative genes of RTT.

The rate of somatic mosaic in males (16.7%, 3/18) was significantly higher than that in females (0.4%, 2/453) (*p* = 0.0004492, odds ratio = 43.47 [95% CI 6.33 – inf] by a two-tailed Fisher’s exact test). Among different studies, 69% (9/13) of reported RTT mosaic patients were male.^8–15^ The application of mosaicism screening had a significant impact on the genetic diagnosis of RTT males. Therefore, when encountering male patients with RTT-related phenotypes, *MECP2* mosaicism should be seriously taken into consideration.

The position p.106Arg was a mosaic hotspot in our cohort. Patient R113, a boy with the c.317G>A, p.(Arg106Gln) variant (MAF = 6.50%) had a phenotype milder than those of the other two patients with p.106Arg variants, i.e., female patient R365 (c.317G>A, p.[Arg106Gln], MAF = 38.08%) and male patient R782 (c.316C>T, p.[Arg106Trp], MAF = 26.32%). Several studies, including ours, have shown that male patients who had variants causing typical RTT in females usually presented with severe neonatal encephalopathy and early death.^15,29^ Male patients with somatic mosaic variants may have milder phenotypes in comparison with those of female patients with the same variants and a heterozygous genotype primarily because of differences between their MAFs. However, the correlation between MAFs and the severity of the disease, as well as the minimum threshold of MAFs, requires further investigation in a larger cohort.

Mosaic variant (c.353G>T, p.[Gly118Val]) in patient R734 has not been reported previously. It is located in a highly conserved methyl-CpG-binding (MBD) domain region, and it was predicted as damaging by MutationTaster, SIFT, and PolyPhen-2. The variant might result in abnormal binding between MeCP2 and its target DNA.^30^ Therefore, mosaic variant (c.353G>T, p.[Gly118Val]) was considered to be the pathogenic cause of RTT in this male patient, although it could be nonpathogenic in females.

More than 99% of RTT cases are sporadic, and their pathogenic *MECP2* variants are mainly de novo and of paternal origin.^16,17^ Therefore, we wondered whether paternal embryonic mosaicism (affecting both somatic and germline tissues) or clonal expansion (germline-specific) mosaicism is the origin of *MECP2* de novo variants in RTT patients. Germline mosaicism in a father with two RTT daughters (half-sisters) was first reported by Evans et al.^20^ No additional findings about germline mosaicism in RTT parents were reported thereafter. This study is the first systematic research assessing paternal germline and somatic mosaicism in RTT families. As a result, no somatic mosaic variants were identified in the peripheral blood of 74 fathers, but, notably, germline *MECP2* mosaicisms were discovered in 5 fathers from 21 families (23.8%, 5/21) with RTT daughters, and the MAFs ranged from 0.03% to 7.55%. The germline mosaicism rate (5/21, 23.8%) was much higher than that of somatic mosaicism (0% for 74 fathers and 1.1% (5/471) for patients). These results indicate that paternal germline-specific mosaicism is an important, and yet underestimated, source of pathogenic gene variation in RTT patients.

In ten fathers, mDDPCR was performed using samples of blood, saliva, hair follicle, urine, and semen. For four of the tested fathers, mosaic variants were found only in the germ cells, indicating that the mosaic variants of these subjects may have occurred during a later embryonic stage and might be present only during self-renewal of primordial germ cells. One father also had somatic mosaicisms in saliva and blood in addition to germline mosaicism, which might have occurred during early embryonic development before the differentiation of germinal layers. For this subject, the MAF in germ cells was higher (7.55%) than that in saliva (0.43%) and blood (0.28%), but the reason for this difference is unknown. It has been reported that mosaic variants accumulate in paternal germline cells with age because of constant meiosis and “selfish spermatogonial selection.”^31^ This type of variant accumulation has been described in several diseases, including Apert syndrome (caused by *FGFR2* pathogenic variants) and Costello syndrome (caused by *HRAS* pathogenic variants).^32,33^ An alternative explanation for high germline mosaicism is that the primordial germ cells of males underwent methylation reprogramming twice during embryonic development, whereas this process occurred only once in other tissues.^34^ Therefore, the genomic DNA of sperm is more unstable than that of other tissues and develops cytidine-to-thymine variation due to spontaneous deamination of methylated CpG. However, the mechanism underlying the high rate of germline mosaicism in comparison with other tissues requires further investigation. The high rate of germline mosaicism observed in this study led us to reconsider the concept of de novo variants; a large number of de novo variants in patients might originate from parental germline mosaicisms.

One father with germline mosaicism was diagnosed with asthenospermia (R831F), while one father nearly met the diagnostic criteria for asthenospermia (R848F, Table 1). Moreover, although almost all male mice of RTT model strains are infertile, including Mecp2-null (stock no. 003890), Mecp2^T158M^ (stock no. 026762), Mecp2^T158A^ (stock no. 004781), Mecp2^R106W^ (stock no. 004781), Mecp2^R306C^ (stock no. 026762), Mecp2^A140V^ (stock no. 016207), and Mecp2^R168X^ (stock no. 006028) (Jackson Laboratory, https://www.jax.org/search?q=Mecp2), the mechanism underlying this phenotype remains unclear. MeCP2 was reported to be involved in spermatogenesis in rats.^35^ Therefore, we proposed the hypothesis that the mutant MeCP2 protein destroys the microenvironment of spermatogenesis. MeCP2 is a very important epigenetic factor that plays crucial roles in transcriptional activation/silencing for hundreds of genes, posttranscriptional modifications, chromosome conformation, processing of noncoding RNA, transposon insertions, and other processes.^36,37^ Therefore, even a relatively small change in MeCP2 might have serious consequences. For example, changes in the microenvironment of spermatogenesis, including disrupted transposons or abnormal RNA retention, may lead to abnormal spermatogenesis. Recent studies demonstrated that massive RNA elimination and inhabitation of transposons are crucial for normal spermatogenesis.^38,39^ Comprehensive functional studies are required to confirm this hypothesis. However, subject R831F has a RTT daughter and a healthy son, whereas subject R848F has a RTT daughter, which suggests that sperm carrying *MECP2* variants might have a spermatogonial selection advantage.

The findings of this study indicate that somatic *MECP2* mosaic variants are partially responsible for the pathogenesis of RTT, especially when they occur in RTT male patients. Identification of germline mosaicisms in the fathers of RTT girls has important implications for genetic counseling in RTT families. Finally, germline mosaicisms also reveal important information about the genetic mechanism underlying the paternal origin bias of RTT.

## Electronic supplementary material

Supplemental Material

Supplemental table S6

Supplemental table S7
