# Supplementary material for: Genomic mosaicism in the pathogenesis and inheritance of a Rett syndrome cohort
Source: Genet Med. 2018 Nov 8;21(6):1330–8. doi: 10.1038/s41436-018-0348-2 (PMC6752670; doi:10.1038/s41436-018-0348-2)
Supplement: Supplementary file 1 — Supplemental Material [file 41436_2018_348_MOESM1_ESM.doc]

**Supplemental Data Contents:**

**Supplemental Figure:**

Figure S1: MAF and 95% binomial confidence intervals measured by micro-droplet digital PCR.

Figure S2: MAFs of different samples tested by mDDPCR.

**Supplemental Tables:**

Table S1: Summary of RTT and RTT-like patients in the entire cohort.

Table S2: 146 RTT and RTT-like patients in whom no *MECP2/CDKL5/FOXG1* pathogenic variants were identified.

Table S3: Relative information of fathers who underwent germline mosaicism detection.

Table S4: Primer sequences used for PASM validation.

Table S5: Ten hotspot variants and two non-hotspot variants of *MECP2* targeted by TaqMan assays.

Table S6: mDDPCR and PASM validation results.

Table S7: Summary of *MECP2, CDKL5* and *FOXG1* variants in probands.

Table S8: Clinical description of five patients with MECP2 mosaic variants.

**Supplemental material 1**


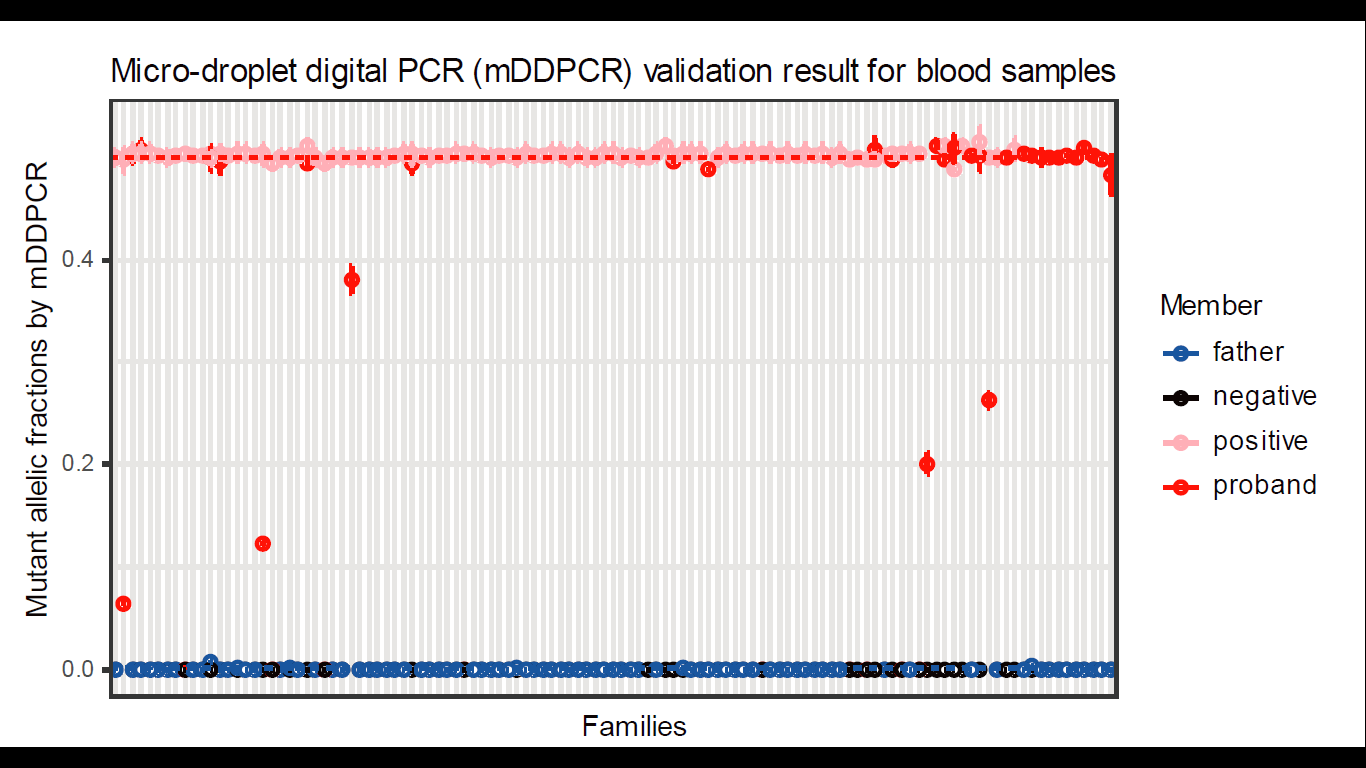


Figure S1: MAF and 95% binomial confidence intervals measured by micro-droplet digital PCR (mDDPCR) in blood samples from 74 fathers who have a RTT daughter with confirmed “*de novo*” pathogenic variants in the *MECP2* gene, as well as 47 patients with candidate mosaic variants in the *MECP2* gene.


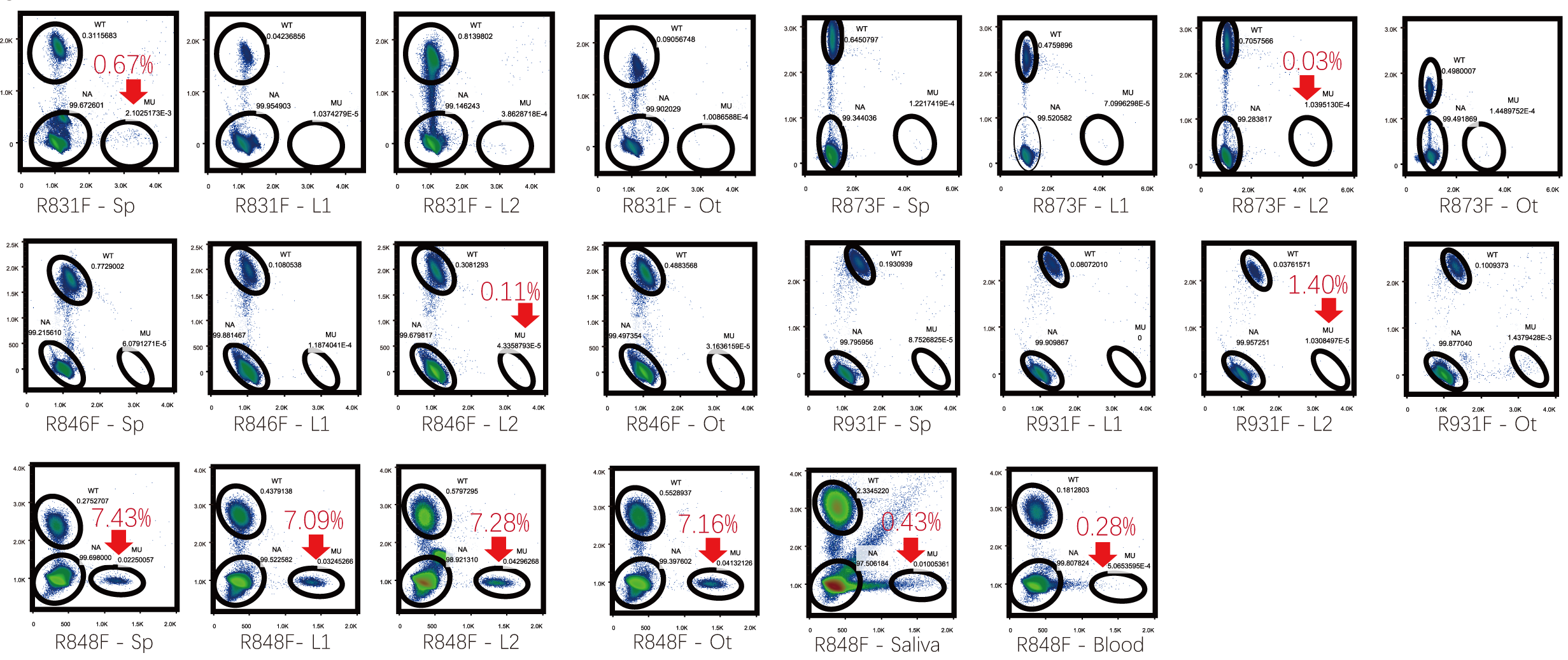


Figure S2. MAFs of different samples tested by mDDPCR.

**Table S1. Summary of RTT and RTT-like patients in the entire cohort.**

| **Groups** | **F** | **M** | **Total** |
| --- | --- | --- | --- |
| Typical RTT | 383 | 6 | 389 |
| Atypical | 48 | 1 | 49 |
| RTT-like | 22 | 11 | 33 |
| Total | 453 | 18 | 471 |

F, female; M, male.

**Table S2. 146 RTT and RTT-like patients in whom no *MECP2/CDKL5/FOXG1* pathogenic variants were identified.**

| **Groups** | **F** | **M** | **Total** |
| --- | --- | --- | --- |
| Typical RTT | 96 | 4 | 101 |
| Atypical RTT | 22 | 1 | 23 |
| RTT-like | 17 | 6 | 23 |
| Total | 135 | 11 | 147 |

F, female; M, male; Han-RTT, Hanefeld variant of RTT.

**Table S3. Relative information of fathers who underwent germline mosaicism detection.**

| **ID** | ***MECP2* variations of proband** | | **multiple peripheral samples** |
| --- | --- | --- | --- |
| R193F | c.316C>T | p.(Arg106Thr) | Yes |
| R382F | c.763C>T | p.(Arg255Ter) | Yes |
| R776F | c.808C>T | p.(Arg270Ter) | No |
| R831F | c.502C>T | p.(Arg168Ter) | No |
| R846F | c.502C>T | p.(Arg168Ter) | No |
| R848F | c.806delG | p.(Gly269Alafs*20) | Yes |
| R854F | c.763C>T | p.(Arg255Ter) | No |
| R863F | c.856_859del | p.(Lys286Profs*2) | No |
| R865F | c.916C>T | p.(Arg306Cys) | No |
| R871F | c.397C＞T | p.(Arg133Cys) | No |
| R873F | c.880C>T | p.(Arg294Ter) | No |
| R874F | c.916C>T | p.(Arg306Cys) | No |
| R877F | c.808C>T | p.(Arg270Ter) | No |
| R895F | c.916C>T | p.(Arg306Cys) | No |
| R915F | c.763C>T | p.(Arg255Ter) | Yes |
| R922F | c.808C>T | p.(Arg270Ter) | Yes |
| R925F | c.502C>T | p.(Arg168Ter) | Yes |
| R931F | c.502C>T | p.(Arg168Ter) | Yes |
| R934F | c.139C>T | p.(Gln47Ter) | Yes |
| R957F | c.473C>T | p.(Thr158Met) | Yes |
| R958F | c.502C>T | p.(Arg168Ter) | Yes |

Multiple peripheral samples included hair follicle, buccal swab, saliva and urine. Blood and semen were obtained from all 21 fathers, and multiple peripheral samples were obtained from 10 fathers.

**Table S4.** Primer sequences used for PASM validation

| **AA change** | **Base change** | **ChrX: pos** | **Forward Primer (5′–3′)** | **Reverse Primer (5′–3′)** |
| --- | --- | --- | --- | --- |
| p.(Arg133Cys) | c.397C>T | 153296882 | CAGGACTTTTCTCCAGGACCC | GGCAGTGTGACTCTCGTTCA |
| p.(Arg168*) | c.502C>T | 153296777 |
| p.(Arg255*) | c.763C>T | 153296516 | ATGGGGAGTACGGTCTCCTG | AGAAACCACCTAAGAAGCCCA |
| p.(Gly269Afs*20) | c.806delG | 153296473 |
| p.(Arg270*) | c.808C>T | 153296471 |
| p.(Lys286Profs*2) | c.856_859del | 153296420 |
| p.(Arg294*) | c.880C>T | 153296399 |
| p.(Arg306Cys) | c.916C>T | 153296363 | GCCGTCGCTCTCCAGTG | CAGGAGACCGTACTCCCCAT |

Reference cDNA: NM_004992.3; reference amino acid sequence: NP_004983.1

**Table S5.** Ten hotspot variants and two non-hotspot variants of *MECP2* targeted by TaqMan assays.

| **Nucleotide change** | **Amino Acid change** | **SNP ID** | **TaqMan assay ID** |
| --- | --- | --- | --- |
| c.316C>T | p.(Arg106Trp) | rs28934907 | AHRSQ7K |
| c.317C>A | p.(Arg106Gln) | - | AHX1JU3 |
| c.353G>T | p.(Gly118Val) | - | AH51857 |
| c.397C>T | p.(Arg133Cys) | rs28934904 | AHMSYIO |
| c.455C>G | p.(Pro152Arg) | rs61748404 | AHN1WOW |
| c.473C>T | p.(Thr158Met) | rs28934906 | AHCTC1V |
| c.502C>T | p.(Arg168Ter) | rs61748421 | AHD2B7P |
| c.880C>T | p.(Arg294Ter) | rs61751362 | AHABGPF |
| c.916C>T | p.(Arg306Cys) | rs28935468 | AHBKEVN |
| c.763C>T | p.(Arg255Ter) | rs61749721 | C_27532119_10 |
| c.860delG | p.(Gly269Afs*20) | rs61750241 | AHPAUU4 |
| c.808C>T | p.(Arg270Ter) | rs61750240 | AHQJS1C |
| Reference cDNA: NM_004992.3  Reference amino acid sequence: NP_004983.1 | |  |  |

**Table S8. Clinical description of five patients with *MECP2*mosaic variants.**

| **Patient** | **R113** | **R365** | **R286** | **R734** | **R782** |
| --- | --- | --- | --- | --- | --- |
| Gender | M | F | F | M | M |
| Age (months) | 52 | 28 | 37 | 30 | 31 |
| Diagnosis | RTT-like | Typical RTT | Typical RTT | Typical RTT | Typical RTT |
| Nucleotide change | c.317G>A | c.316C>T | c.502C>T | c.353G>T | c.316C>T |
| Amino acid change | p. Arg106Gln | p. Arg106Trp | p. Arg168Ter | p. Gly118Val | p. Arg106Trp |
| Mutant allele fraction | 6.50% | 38.08% | 12.28% | 20.11% | 26.32% |
| Regression | N | Y, 17 mo | Y, 15 mo | Y, 13 mo | Y, 18 mo |
| Loss of hand skill | N | Y, 19 mo | Y, 19 mo | Y, 12 mo | No hand use |
| Loss of speech | No speech | Y, 17 mo | Y, 17 mo | Y, 13 mo | No speech |
| Abnormal gait | Y | Y | Unable to walk | Unable to walk | Unable to walk |
| Stereotypic hand acts | Y, 30 mo | Y, 19 mo | Y, 19 mo | Y, 12 mo | Y, 15 mo |
| Breathing disturbance | N | Y, 22 mo | Y, 22 mo | Y, 18 mo | N |
| Bruxism | Y, 36 mo | Y, 22 mo | Y, 22 mo | Y, 18 mo | Y, 30 mo |
| Sleeping disturbance | N | N | N | N | N |
| Abnormal muscular tone | Y | Y | Y | Y | Y |
| Vasomotor disturbance | N | Y | Y | Y | N |
| Scoliosis/kyphosis | N | N | N | N | N |
| Growth retardation | Y | Y | Y | Y | Y |
| Small cold hand/feet | N | Y | Y | Y | Y |
| Laughing/screaming spells | N | N | N | N | N |
| Diminished response to pain | N | N | Y | Y | Y |
| Intense eye communication | N | N | N | N | N |
| Seizures | N | Y, 24 mo | N | N | N |

M, male; F, female; mo, months; AA, amino acid; N, no; Y, yes.

**Data deposition:**

mDDPCR data are available at https://pan.baidu.com/s/1bFndBqHcuASlH98Q1fTi7A under accession code q4mq
